# Severe Illness Associated with Reported Use of Synthetic Marijuana — Colorado, August–September 2013

**Published:** 2013-12-13

**Authors:** Tista Ghosh, Rachel Herlihy, Mike Van Dyke, Stephanie Kuhn, Burrer Sherry, Melissa Halliday, Bridget Spelke, Tesfaye Bayleyegn, Amy Wolkin, Lauren S. Lewis, Ethan Fechter-Leggett, Olaniyi Olayinka

**Affiliations:** Colorado Dept of Health and Environment; Div of Environmental Hazards and Health Effects, National Center for Environmental Health; EIS Officer, CDC

On August 30, 2013, the Colorado Department of Public Health and Environment (CDPHE) was notified by several hospitals of an increase in the number of patients visiting their emergency departments (EDs) with altered mental status after using synthetic marijuana. Synthetic marijuana is dried plant material sprayed with various synthetic cannabinoids and smoked as an alternative to smoking marijuana. In response to the increase in ED visits associated with the use of synthetic marijuana, CDPHE asked all Colorado EDs to report through EMResource (a web-based reporting system) any patients examined on or after August 21 with altered mental status after use of a synthetic marijuana product. Serum and urine specimens from patients also were requested. On September 8, CDPHE, with the assistance of CDC, began an epidemiologic investigation to characterize the outbreak, determine the active substance and source of the synthetic marijuana product, and prevent further morbidity and mortality. Investigators reviewed ED visit reports submitted through EMResource and medical charts. A probable case was defined as any illness resulting in a visit to a Colorado ED during August 21–September 18, 2013, by a patient with suspected synthetic marijuana use in the 24 hours preceding illness onset. Of 263 patient visits reported to CDPHE through EMResource (214) and other means, such as e-mail and fax (49), a total of 221 (84%) represented probable cases (Figure).

Among the 221 probable cases, abstracted medical records from a convenience sample of 127 (58%) patients were used for descriptive study. Median age of the 127 patients was 26 years (range: 13–60 years), and 101 (80%) were male. Clinical signs and symptoms included systolic blood pressure >120 mmHg in 81 (64%), heart rate >100 beats per minute in 73 (57%), somnolence in 45 (35%), aggressive or violent behavior in 40 (32%), agitation in 40 (32%), and confusion in 32 (25%).

Of the 127 patients, a total of 111 (87%) were treated and discharged from the ED. Sixteen (13%) were admitted, 10 of whom were admitted to an intensive care unit. No deaths were reported among the 127 patients. All 127 patients were reported from EDs in the Denver metropolitan area (99) or Colorado Springs (28).

Brand names of synthetic marijuana products that investigators determined had been used by the patients included Black Mamba, Crazy Monkey, Crazy Clown, Dead Man Walking, Funky Monkey, Sexy Monkey, SinX, Spice, TenX, Twilight, and 3X. Patients also identified two convenience stores, one “head shop,” and one gas station as sources of synthetic marijuana products involved in this outbreak. These stores subsequently were closed by Colorado law enforcement officials. To alert the public to the outbreak, CDPHE released messages regarding the dangers of synthetic marijuana via social media and the news media. The investigation provided geographic and demographic information that enabled CDPHE to focus the messaging toward teens and young men in certain geographic areas.

Although the clinical features observed in patients were consistent with synthetic marijuana exposure described in the medical literature (2,3), no standard laboratory tests are available to confirm synthetic marijuana intoxication. Currently, CDPHE is coordinating with the Colorado Bureau of Investigation to determine whether two new variants of synthetic marijuana, ADBICA (N-(1-amino-3,3-dimethyl-1-oxobutan-2-yl)-1-pentyl-1H-indole-3-carboxamide) and ADB-PINACA (N-(1-amino-3,3-dimethy-1-oxobutan-2-yl)-1-pentyl-1H-indazole-3-carboxamide), that were found in products seized by the Colorado Bureau of Investigation shortly before the outbreak, contributed to the illnesses.

ADB-PINACA was linked to a similar outbreak in Georgia in August 2013 (4). The public should be aware of the potential dangers of synthetic marijuana use, and EDs and public health departments should remain vigilant for reports of adverse health effects from synthetic marijuana use so that they can detect outbreaks more readily and monitor the effectiveness of prevention efforts.

## Figures and Tables

**FIGURE:**
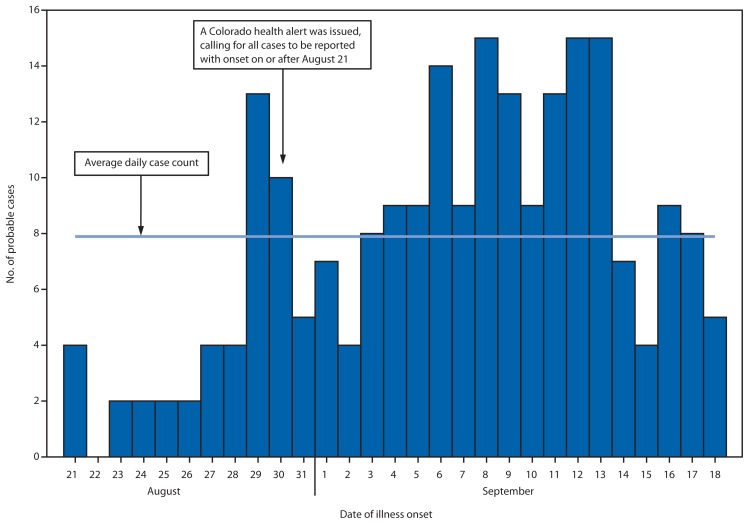
Number of probable cases (n = 221) of severe illness associated with use of synthetic marijuana, by date of illness onset — Colorado, August 21–September 18, 2013
